# Vacuum induced transparency and photon number resolved Autler-Townes splitting in a three-level system

**DOI:** 10.1038/s41598-018-22666-2

**Published:** 2018-03-14

**Authors:** Jiang-hao Ding, Sai-nan Huai, Hou Ian, Yu-xi Liu

**Affiliations:** 1 Institute of Microelectronics, Tsinghua University, Beijing, 100084 China; 2 Institute of Applied Physics and Materials Engineering, University of Macau, 999078 Macau, China; 3 Tsinghua National Laboratory for Information Science and Technology (TNList), Beijing, 100084 China

## Abstract

We study the absorption spectrum of a probe field by a Λ-type three-level system, which is coupled to a quantized control field through the two upper energy levels. The probe field is applied to the ground and the second excited states. When the quantized control field is in vacuum, we derive a threshold condition to discern vacuum induced transparency (VIT) and vacuum induced Autler-Townes splitting (ATS). We also find that the parameter changing from VIT to vacuum induced ATS is very similar to that from broken *PT* symmetry to *PT* symmetry. Moreover, we find the photon number resolved spectrum in the parameter regime of vacuum induced ATS when the mean photon number of the quantized control field is changed from zero (vacuum) to a finite number. However, there is no photon number resolved spectrum in the parameter regime of VIT even that the quantized control field contains the finite number of photons. Finally, we further discuss possible experimental realization.

## Introduction

Electromagnetically induced transparency (EIT)^1–4^ is extensively studied in various three-level systems^5,6^, which interact with a classical control field and a classical probe field. If a medium can be modeled as an ensemble of many identical three-level atoms, then using EIT mechanism and under appropriate conditions, we can find that the absorption of the probe field by the medium can be reduced, and the medium can become effectively transparent at the zero absorption for the probe field, e.g., the probe field can freely pass through the medium. Thus, EIT is usually used to eliminate the medium effect on the probe field by a classical control field. If the absorption is monitored, then the reduction of the absorption is characterized by a dip in the absorption spectrum, and also there are two peaks surrounding the dip.

It is known that EIT is closely related to Autler-Townes splitting (ATS)^7^. Both EIT and ATS study the absorption of the probe field by a driven three-level system. Both of them display a reduction in absorption, and have two peaks and a dip in their absorption spectra^8^. However, the physical mechanisms of their peaks and dip are different. In ATS, the peaks and dip are due to two resonances corresponding to energy level splitting, induced by the strong control field. However, the peaks and dip in EIT are due to the quantum interference when two resonances in ATS become overlapped. The quantum interference can be understood via dressed states^9^, formed by the three-level system and control field. That is, one resonant transition path, linked by the probe field in bare atomic energy levels when the control field is not applied, becomes into two resonant transition paths in dressed state picture when the control field is applied, and thus the resonant absorption of the probe field in bare atom might be canceled through the quantum interference of transitions from these two paths. The threshold condition to distinguish EIT from ATS has been theoretically explored^10,11^ and also experimentally studied^12–15^. ATS occurs when the strength of the control field is larger than the critical value determined by the decay rates of the three-level systems. EIT may appear when the strength of the control field is smaller than the critical value. Roughly speaking, the strength of the control field for EIT is smaller than that for ATS. A natural question is whether two peaks and a dip can still appear when the photon number in the control field is finite or is further reduced to zero? The answer to this question is related to the interaction between the quantized control field and the three-level system.

We know that quantized fields can also form dressed states with atoms. However, dressed states composed by quantized field and atom are very different from those composed by classical field and atom. For example, a dressed two-level system by a classical field still possesses a character of two energy levels^9,16^, which was experimentally demonstrated^17^, for example, via two-level superconducting quantum circuits^18–21^. Whereas, a dressed two-level system by a quantized field possesses many energy levels. Using a structure of three energy levels, chosen from many energy levels of dressed two-level systems by a quantized field^22^, both EIT and ATS have been theoretically studied^23,24^. Moreover, dressed two-level systems by quantized fields are also used to generate stimulated amplification^25^, demonstrate attenuation effects^26^, and exhibit polariton states with selective radiation spectrum^27^.

Let us come back to three-level system with Λ transitions for EIT and ATS. If the classical control field in such system is replaced by a quantized control field, then the so-called vacuum induced transparency (VIT), resulted from the quantum interference has been theoretically and experimentally studied when the quantized control field is in vacuum and the coupling strength between three-level system and quantized control field is in the weak coupling regime^28,29^. However, to our knowledge, the energy-level splitting induced by vacuum is not studied when such three-level system and quantized control field are in the strong coupling regime. Hereafter, we call vacuum induced energy level splitting as vacuum induced ATS in analogy to ATS. Furthermore, the threshold condition to discern VIT from vacuum induced ATS is not studied. Although the photon number effect of the quantized control field on the quantum interference is mentioned in^29^, and also the dependence of the EIT group delay on the photon number is studied in^30^, there is no study about the photon number effect on the energy level splitting in analogy to ATS.

Here, we study the absorption spectrum of a probe field by a Λ-type three-level system, which is coupled to a quantized control field through the first and second excited states. When the quantized control field is in vacuum, we first derive a threshold condition to discern VIT from vacuum induced ATS, then we further analyze how VIT is changed to vacuum induced ATS when the decay rate of the cavity field or the coupling strength between the quantized control field and three-level system is varied. This analysis is very similar to that for *PT* symmetric systems, e.g., in^31–33^. When the quantized control field contains finite number of photons, we first show how the photon number affects the absorption spectrum when the coupling strength is in the VIT parameter regime. In particular, we will show that the photon number resolved spectrum can be found when the coupling strength is in the parameter regime of vacuum induced ATS. In view of experimental progresses of superconducting quantum devices^18–21^ and circuit quantum electrodynamics (CQED)^34^, on which ATS^35–42^, population trap^43^, adiabatic population transfer^44^, and EIT^45,46^ have been theoretically and experimentally demonstrated, we will also discuss possible experimental realization of VIT and vacuum induced ATS in superconducting CQED systems.

In this paper, we first describe theoretical model and write out dressed states of the studied system. Then we give a definition of the susceptibility, and show detailed steps for deriving a formula to describe the susceptibility. We analyze the properties of the absorption spectrum via susceptibility when the quantized control field is in vacuum. Similar to the asymmetric profile of the absorption spectrum^47,48^ for classically driven three-level system, we give a detailed analysis on the asymmetric profile of two resonances. We further derive a threshold condition to discern VIT from vacuum induced ATS. We also analyze photon number effect on EIT and ATS by incoherently or coherently pumping the quantized control field. In particular, we show the photon number resolved spectrum when the coupling strength between quantized control field and three-level system is in the parameter regime of vacuum induced ATS. We will also analyze the reason why there is no photon number resolved spectrum when the coupling strength between the quantized controlled field and three-level system is in the parameter regime of VIT. We apply our study to atomic systems or superconducting CQED systems and discuss possible experimental realization. Finally, we summarize the results.

## Hamiltonian of the system

**Figure 1:**
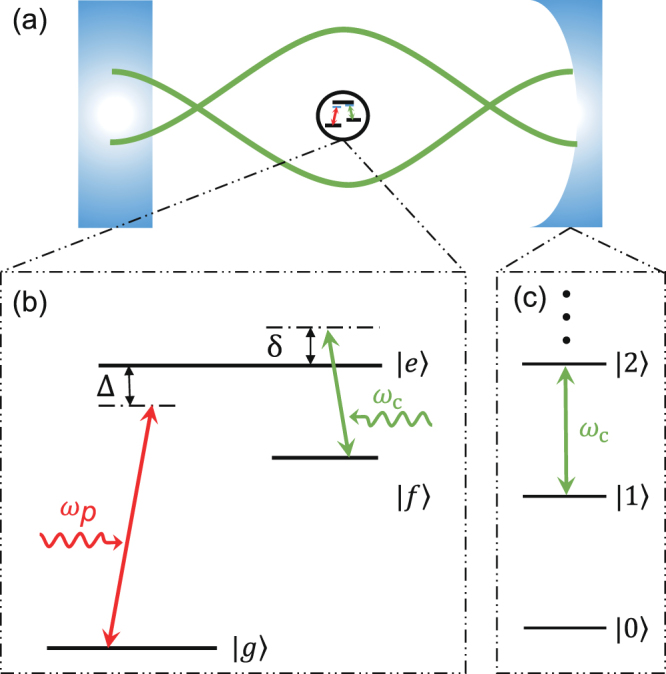
(**a**) A schematic diagram for a three-level system with Λ-type transitions inside the cavity. Here two green curves schematically represent the cavity field. (**b**) The schematic diagram for three-level system coupled to a single-mode cavity field and a classical probe field. The cavity field induces the transition between the energy levels |*e*〉 and |*f*〉, however the probe field induces the transition between the energy levels |*e*〉 and |*g*〉. Here, Δ = *ω*_*e*_ − *ω*_*p*_ is the detuning between the frequency *ω*_*p*_ of the probe field and the transition frequency *ω*_*e*_ of the three-level system, *δ* = *ω*_*c*_ − (*ω*_*e*_ − *ω*_*f*_) denotes the detuning between the frequency *ω*_*c*_ of the cavity field and the transition frequency *ω*_*e*_ − *ω*_*f*_ of the three-level system. (**c**) A schematic diagram for the energy levels of the cavity field with the equal energy levels spacing *ω*_*c*_.

As schematically shown in Fig. 1, we study a Λ-type three-level system, which is placed inside a cavity. The ground state, first and second excited states of the three-level system are denoted by |*g*〉, |*f*〉 and |*e*〉. For generality of the study, we first do not specify this three-level system to a particular physical object. We further assume that the quantized single-mode cavity field with frequency *ω*_*c*_ induces the transition from the energy level $|f\rangle$ to the energy level $|e\rangle$, while a weak classical probe field with frequency *ω*_*p*_ induces the transition between the energy levels $|g\rangle$ and $|e\rangle$. Later on, we call the quantized single-mode cavity field as the quantized control field or the cavity field. Under the rotating-wave approximation, the Hamiltonian of the whole system is given by

1 $$H={H}_{c}+{H}_{p},$$

with

2 $${H}_{c}=\hslash {\omega }_{c}{a}^{\dagger }a+\hslash {\omega }_{e}|e\rangle \langle e|+\hslash {\omega }_{f}|f\rangle \langle f|+\hslash \eta (|e\rangle \langle f|a+{\rm{h}}{\rm{.c}}\mathrm{.),}$$

and

3 $${H}_{p}=\hslash \varepsilon (|e\rangle \langle g|{e}^{-i{\omega }_{p}t}+{\rm{h}}{\rm{.c}}\mathrm{.),}$$

where *ω*_*f*_ and *ω*_*e*_ are the transition frequencies from the first excited state $|f\rangle$ and the second excited state $|e\rangle$ to the ground state |*g*〉, respectively. The parameter *η* denotes the coupling strength between the three-level system and the cavity field, *ε* denotes the coupling strength between the probe field and the three-level system.

It is obvious that the probe field can be resonantly absorbed by the three-level system when the cavity field is not coupled to the three-level system, i.e., *η* = 0. To clearly show the effect of the cavity field on the absorption of the probe field, we now rewrite the Hamiltonian in Eq. (2) in the dressed state basis, formed by the cavity field and two upper energy levels |*e*〉 and |*f*〉 of the three-level system. That is, the Hamiltonian in Eq. (2) can be rewritten as

4 $${H}_{c}=\sum _{n}({E}_{-,n}|{u}_{n}\rangle \langle {u}_{n}|+{E}_{+,n}|{v}_{n}\rangle \langle {v}_{n}|),$$

in the dressed state basis |*u*_*n*_〉 and |*v*_*n*_〉, given by

5 $$|{u}_{n}\rangle =\,\cos \,{{\theta }}_{n}|n,e\rangle -\,\sin \,{{\theta }}_{n}|n+1,\,f\rangle ,$$

6 $$|{v}_{n}\rangle =\,\sin \,{{\theta }}_{n}|n,e\rangle +\,\cos \,{{\theta }}_{n}|n+1,f\rangle ,$$

with *n*-dependent parameter

7 $${{\theta }}_{n}=\frac{1}{2}{\tan }^{-1}[\frac{2\eta \sqrt{n+1}}{\delta }],$$

and *δ* = *ω*_*c*_ − (*ω*_*e*_ − *ω*_*f*_). Here, the state |*n* + 1, *f*〉 (or |*n*, *e*〉) denotes that there are *n* + 1 (or *n*) photons inside the cavity and the three-level system is in the state |*f*〉 (or |*e*〉). Later on, for convenience, we call |*u*_*n*_〉 and |*v*_*n*_〉 as *n*-photon dressed states. The eigenvalues *E*_−,*n*_ and *E*_+, *n*_, corresponding to eigenstates |*u*_*n*_〉 and |*v*_*n*_〉, are given by

8 $${E}_{\pm ,n}=(n+\frac{1}{2})\hslash {\omega }_{c}+\frac{\hslash }{2}({\omega }_{e}+{\omega }_{f})\pm \frac{\hslash }{2}\sqrt{{\delta }^{2}+4{\eta }^{2}(n+\mathrm{1)}}\mathrm{.}$$

**Figure 2:**
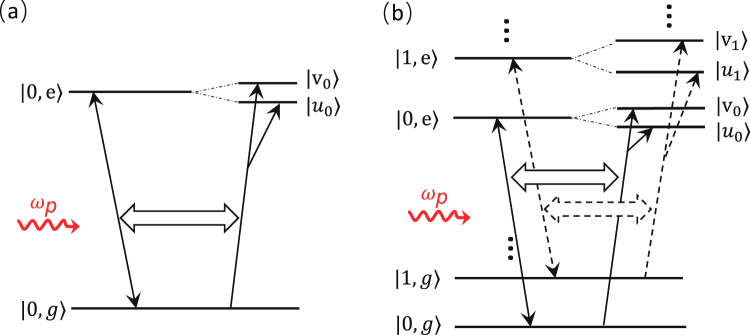
Schematic diagrams for the coupling between the dressed three-level system and the weak probe field. (**a**) shows that the probe field, used to induce the transition between the ground state $|g\rangle$ and the excited state $|e\rangle$ when the cavity field does not exist, is changed to induce the transitions from the ground state $|0,\,g\rangle$ to the states $|{u}_{0}\rangle$ and $|{v}_{0}\rangle$, respectively, in the dressed state basis when the cavity field is in vacuum. (**b**) shows that the transition from the state $|n,g\rangle$ to the state $|n,e\rangle$ are changed to the transitions from the state $|n,g\rangle$ to the states $|{u}_{n}\rangle$ and $|{v}_{n}\rangle$, respectively, in the dressed state basis when the cavity field contains the finite number of photons. Here, as an example, we just take *n* = 0 and *n* = 1.

Using dressed states in Eqs (5) and (6) as basis, the Hamiltonian *H*_*p*_ in Eq. (3) can be rewritten as

9 $${H}_{p}=\hslash \varepsilon \sum _{n}[(\cos \,{{\theta }}_{n}|{u}_{n}\rangle +\,\sin \,{{\theta }}_{n}|{v}_{n}\rangle )\langle n,g|{e}^{-i{\omega }_{p}t}+{\rm{h}}{\rm{.c}}{\rm{.}}],$$

by replacing |*n*, *e*〉 with $|{u}_{n}\rangle$ and $|{v}_{n}\rangle$. We note that $|n,g\rangle$ is not a dressed state, but it is orthogonal to $|{u}_{n}\rangle$ and $|{v}_{n}\rangle$. This is because $|{u}_{n}\rangle$ and $|{v}_{n}\rangle$ are linear superpositions of $|n,e\rangle$ and $|n+1,\,f\rangle$, which are orthogonal to $|n,g\rangle$. Here, we will study the absorption spectrum of the whole system to the probe field when the cavity field is in vacuum or contains the finite number of photons, as schematically shown in Fig. 2. The basic mechanism of absorption for two cases can be qualitatively explained as below.

If there is no photon inside the cavity or the cavity field is in vacuum, then all populations are in the ground state $|0,\,g\rangle$ when the whole system reaches the steady state. Thus, the probe field, which induces the transition between the states $|g\rangle$ and $|e\rangle$ when there is no cavity field, is changed to link the transition from the state $|0,\,g\rangle$ to the state $|0,\,e\rangle$ when the cavity field is coupled. Thus, in the dressed state basis, as schematically shown in Fig. 2(a), one transition path from the state $|0,\,g\rangle$ to the state $|0,\,e\rangle$ is changed to two transition paths from the state $|0,\,g\rangle$ to the states $|{u}_{0}\rangle$ and $|{v}_{0}\rangle$, respectively. This is because $|0,\,e\rangle$ can be expressed as the superposition of $|{u}_{0}\rangle$ and $|{v}_{0}\rangle$. In different parameter regimes of the coupling strength between the quantized control field and the three-level system, these transitions might result in either VIT spectrum^28,29^ or vacuum induced ATS spectrum. In the following, we will give a detailed study for a threshold condition to discern them.

If there is the finite number *N* of photons inside the cavity, all states $|n,\,g\rangle$, $|n,\,f\rangle$ and $|n,\,e\rangle$ with $n=0,1,\cdots ,\,N$ might be occupied with the certain probability when the whole system reaches the steady state. Thus, the probe field, which induces the transitions between the energy levels |*g*〉 and |*e*〉 when there is no cavity field, links *N* transitions between the energy levels |*n*, *g*〉 and |*n*, *e*〉 ($n=0,1,\cdots ,\,N$) when the cavity field is coupled. In the dressed state basis, as schematically shown in Fig. 2(b), each probe-field-induced transition from the state |*n*, *g*〉 to the state |*n*, *e*〉 is changed to transitions from the state |*n*, *g*〉 to the dressed states $|{u}_{n}\rangle$ and $|{v}_{n}\rangle$, respectively. In this case, the absorption spectrum of the probe field should be sum of 2*N* transitions from the state |*n*,*g*〉 to the dressed states $|{u}_{n}\rangle$ and $|{v}_{n}\rangle$ with $n=0,1,\cdots ,\,N$. These 2*N* transitions play different roles in the parameter regime of VIT and vacuum induced ATS. The quantitative analysis will be given below.

### Susceptibility and master equation

The key parameter to characterize the absorption of a probe field by a system is the linear susceptibility *χ*. Its real and imaginary parts represent the dispersion and the absorption of the probe field, respectively. When the probe field is applied to a three-level system via the transition from the state |*g*〉 to the state |*e*〉, the linear susceptibility *χ* of the three-level system is given as

10 $$\chi =\frac{{\mu }_{ge}}{{\varepsilon }_{0}\varepsilon }{\rho }_{ge},$$

where *μ*_*ge*_, *ε*_0_, and *ε* are the dipole moment from the state |*g*〉 to the state |*e*〉 of the three-level system, the vacuum permittivity, and the coupling strength between the probe field and the three-level system. Thus, *χ* is determined by *ρ*_*ge*_. When the quantized control field is coupled to the tree-level system, the matrix element *ρ*_*ge*_ in Eq. (10) can be further expressed as

11 $${\rho }_{ge}=\sum _{n=0}^{\infty }\langle n,g|\rho |e,n\rangle \mathrm{.}$$

We first show how to obtain *ρ*_*ge*_ by solving the master equation

12 $$\dot{\rho }=\frac{1}{i\hslash }[H,\rho ]+\kappa \mathrm{(2}a\rho {a}^{\dagger }-{a}^{\dagger }a\rho -\rho {a}^{\dagger }a)+\sum _{i,j=e,f,g}^{i\geqslant j}\frac{{\gamma }_{ij}}{2}\mathrm{[2}{\sigma }_{ji}\rho {\sigma }_{ij}-{\sigma }_{ij}{\sigma }_{ji}\rho -\rho {\sigma }_{ij}{\sigma }_{ji}],$$

for the reduced density matrix *ρ* of the system in the Born-Markov approximation when the system is at zero temperature. We notice that incoherent pumping (the finite temperature effect) or coherent pumping to the quantized control field will be studied in later of this paper. Here, the Hamiltonian *H* is given by Eq. (1). In Eq. (12), ${\sigma }_{ij}=|i\rangle \langle j|$ is the ladder operator of the three-level system, where $|i\rangle$ and $|j\rangle$ are one of the states $|g\rangle$, $|f\rangle$, and $|e\rangle$ with the order from the ground to the second excited state, *γ*_*ij*_ denotes the decay rate of the three-level system when the cavity field is not coupled to the three-level system. For example, *γ*_*eg*_ denotes the decay rate from the state |*e*〉 to the state |*g*〉 when the cavity field is not coupled to the three-level system. *κ* represents the decay rate of the quantized single-mode cavity field.

The absorption spectrum of the probe field by quantized-field-controlled three-level system can be more conveniently solved in dressed state basis. Thus, using the states |*n*, *g*〉, the dressed states |*u*_*n*_〉 and |*v*_*n*_〉 in Eqs (5) and (6), formed by states $|n,\,e\rangle ,\,|n+\mathrm{1,}\,f\rangle$ of the whole system as the basis, the operators *σ*_*ij*_, *a*^†^ and their hermitian conjugations in the master eq. (12) can be rewritten via the relations

13 $${\sigma }_{ij}=|i\rangle \langle j|=\sum _{n}|n,i\rangle \langle j,n|,$$

14 $${a}^{\dagger }=\sum _{n,i}\sqrt{n+1}|n+\mathrm{1,}\,i\rangle \langle i,\,n|\mathrm{.}$$

We note that the completeness conditions ∑_*n*_|*n*〉〈*n*| = 1 and ∑_*i*_|*i*〉〈*i*| = 1 for the states |*n*〉 and |*i*〉 of the single-mode cavity field and three-level system are used when Eqs (13) and (14) are derived. Substituting expressions of the Hamiltonian *H*, *a*^†^, *σ*_*ij*_ and their hermitian conjugations in dressed state basis into Eq. (12), we can have equations of motion for the matrix elements in dressed state basis.

At the zero temperature as shown in Eq. (12), the whole system is in vacuum and only the ground |0, *g*〉 is populated when the whole system is in the steady state. As discussed in Fig. 2(a), the study for the absorption spectrum of the probe field is limited to the subspace formed by three basis states $|0,\,g\rangle ,|0,\,e\rangle ,|1,\,f\rangle$, which can be rewritten in terms of $|0,\,g\rangle \equiv |G\rangle$, $|{u}_{0}\rangle =\,\cos \,{{\theta }}_{0}\mathrm{|0},\,e\rangle -\sin \,{{\theta }}_{0}\mathrm{|1},\,f\rangle \equiv |u\rangle$ and $|{v}_{0}\rangle =\,\sin \,{{\theta }}_{0}\mathrm{|0,}\,e\rangle +\,\cos \,\,{{\theta }}_{0}\mathrm{|1},\,f\rangle \equiv |v\rangle$ in the dressed state basis. Thus the equations of motion for matrix elements *ρ*_*Gv*_ and *ρ*_*Gu*_ are given by

15 $${\dot{\rho }}_{Gv}=\frac{1}{i\hslash }[-{E}_{+\mathrm{,0}}{\rho }_{Gv}+({\rho }_{vv}-{\rho }_{GG})\hslash \varepsilon \,\sin \,{{\theta }}_{0}{e}^{i{\omega }_{p}t}+\hslash \varepsilon \,\cos \,{{\theta }}_{0}{e}^{i{\omega }_{p}t}{\rho }_{uv}]+{{\rm{\Gamma }}}_{{\rm{c}}}{\rho }_{Gu}+{{\rm{\Gamma }}}_{Gv}{\rho }_{Gv},$$

16 $${\dot{\rho }}_{Gu}=\frac{1}{i\hslash }[-{E}_{-\mathrm{,0}}{\rho }_{Gu}+({\rho }_{uu}-{\rho }_{GG})\hslash \varepsilon \,\cos \,{{\theta }}_{0}{e}^{i{\omega }_{p}t}+\hslash \varepsilon \,\sin \,{{\theta }}_{0}{e}^{i{\omega }_{p}t}{\rho }_{vu}]+{{\rm{\Gamma }}}_{{\rm{c}}}{\rho }_{Gv}+{{\rm{\Gamma }}}_{Gu}{\rho }_{Gu}\mathrm{.}$$

Here, we define the relaxation rates Γ_*Gv*_ and Γ_*Gu*_ as

$${{\rm{\Gamma }}}_{Gv}=-{\gamma }_{e}{\sin }^{2}{{\theta }}_{0}-({\gamma }_{f}+\kappa ){\cos }^{2}{{\theta }}_{0},$$

$${{\rm{\Gamma }}}_{Gu}=-{\gamma }_{e}{\cos }^{2}{{\theta }}_{0}-({\gamma }_{f}+\kappa ){\sin }^{2}{{\theta }}_{0},$$

and the relaxation rate

$${{\rm{\Gamma }}}_{{\rm{c}}}=(-{\gamma }_{e}+{\gamma }_{f}+\kappa )\,\sin \,{{\theta }}_{0}\,\cos \,{{\theta }}_{0},$$

with *γ*_*e*_ = (*γ*_*eg*_ + *γ*_*ef*_ + *γ*_*ee*_)/2 and *γ*_*f*_ = (*γ*_*fg*_ + *γ*_*ff*_)/2. Here, we assume *γ*_*gg*_ = 0.

By solving Eqs (15) and (16) via perturbation theory, we can obtain the density matrix *ρ*_*ge*_ in vacuum case, and then obtain the susceptibility *χ* for discussing VIT and vacuum induced ATS by virtue of the imaginary part I*m*[*χ*].

## Vacuum induced transparency and Autler-Townes splitting

### Symmetric or asymmetric absorption

At the zero temperature, the quantized control field is in vacuum and the occupation is only in the ground state |0, *g*〉 when the whole system reaches the steady state. In this case, the susceptibility *χ* is proportional to the matrix element

17 $${\rho }_{ge}=\langle \mathrm{0,}\,g|\rho |e,\,0\rangle =\,\cos \,{{\theta }}_{0}{\rho }_{Gu}+\,\sin \,{{\theta }}_{0}{\rho }_{Gv},$$

which is expressed in the zero-photon dressed state basis. When Eq. (17) is derived, we express |0, *e*〉 as the superposition of zero-photon dressed states |*u*_0_〉 ≡ |*u*〉 and |*v*_0_〉 ≡ |*v*〉, that is, |0, *e*〉 = cos*θ*_0_|*u*〉 + sin*θ*_0_|*v*〉.

It is obvious that *ρ*_*ge*_ can be straightforwardly obtained by solutions of *ρ*_*Gu*_ and *ρ*_*Gv*_, which can be given by solving Eqs (15) and (16) using perturbation theory for different orders of the strength *ε* of the probe field, i.e., ${\rho }_{ij}={\sum }_{m\mathrm{=0}}{\varepsilon }^{m}{\rho }_{ij}^{(m)}$ with the subscript *ij* = *Gv* or *ij* = *Gu*. The zero-order solution ${\rho }_{ij}^{\mathrm{(0)}}$ is a steady state solution of the system when the probe field is not applied to the system. ${\rho }_{ij}^{\mathrm{(0)}}$ can be obtained by solving Eqs (15) and (16) with assumption ∂*ρ*_*ij*_/∂*t* = 0 and *ε* = 0. Using the dressed state basis, we obtain ${\rho }_{GG}^{\mathrm{(0)}}\approx 1$ and ${\rho }_{uu}^{\mathrm{(0)}}={\rho }_{vv}^{\mathrm{(0)}}={\rho }_{uv}^{\mathrm{(0)}}=0$. Substituting these values of the matrix elements into Eqs (15) and (16), and then solving the equations of motion up to the first order of *ε*, we have

18 $${\rho }_{Gv}=\frac{i\varepsilon \{\sin \,{{\theta }}_{0}[i({\omega }_{-\mathrm{,0}}-{\omega }_{p})+{{\rm{\Gamma }}}_{Gu}]-\,\cos \,{{\theta }}_{0}{{\rm{\Gamma }}}_{{\rm{c}}}\}}{{{\rm{\Gamma }}}_{{\rm{c}}}^{2}-[i({\omega }_{-\mathrm{,0}}-{\omega }_{p})+{{\rm{\Gamma }}}_{Gu}][i({\omega }_{+\mathrm{,0}}-{\omega }_{p})+{{\rm{\Gamma }}}_{Gv}]},$$

19 $${\rho }_{Gu}=\frac{i\varepsilon \{\cos \,{{\theta }}_{0}[i({\omega }_{+\mathrm{,0}}-{\omega }_{p})+{{\rm{\Gamma }}}_{Gv}]-\,\sin \,{{\theta }}_{0}{{\rm{\Gamma }}}_{{\rm{c}}}\}}{{{\rm{\Gamma }}}_{{\rm{c}}}^{2}-[i({\omega }_{-\mathrm{,0}}-{\omega }_{p})+{{\rm{\Gamma }}}_{Gu}][i({\omega }_{+\mathrm{,0}}-{\omega }_{p})+{{\rm{\Gamma }}}_{Gv}]},$$

where ${\omega }_{\pm \mathrm{,0}}={E}_{\pm \mathrm{,0}}/\hslash$. Combining Eqs (18, 19) with Eq. (17), we obtain the analytic solution of *ρ*_*ge*_ as

20 $${\rho }_{ge}=\frac{\varepsilon [{\rm{\Delta }}+i({\gamma }_{f}+\kappa )+\delta /\mathrm{2]}}{-{{\rm{\Delta }}}^{2}-i({\gamma }_{e}+{\gamma }_{f}+\kappa ){\rm{\Delta }}+C},$$

with the detuning

21 $${\rm{\Delta }}=\frac{1}{2}({\omega }_{+\mathrm{,0}}+{\omega }_{-\mathrm{,0}})-{\omega }_{p}\mathrm{.}$$

The parameter *C* is given by

22 $$C={\eta }^{2}+{\gamma }_{e}{\gamma }_{f}+{\gamma }_{e}\kappa +\frac{{\delta }^{2}}{4}+i\frac{\delta }{2}(-{\gamma }_{e}+{\gamma }_{f}+\kappa \mathrm{).}$$

We use Eqs (20) and (10) to obtain the imaginary part Im[*χ*] of the susceptibility *χ* as

23 $${\rm{Im}}[\chi ]=Z[{\gamma }_{e}{({\rm{\Delta }}+\frac{\delta }{2})}^{2}+{\eta }^{2}({\gamma }_{f}+\kappa )+{\gamma }_{e}{({\gamma }_{f}+\kappa )}^{2}],$$

which has equivalent form to the imaginary part of the susceptibility in^29^. Here

$$Z=\frac{{\mu }_{ge}}{{\varepsilon }_{0}}\{{(-{{\rm{\Delta }}}^{2}+{\eta }^{2}+{\gamma }_{e}{\gamma }_{f}+\frac{{\delta }^{2}}{4}+{\gamma }_{e}\kappa )}^{2}+{{[-{\rm{\Delta }}({\gamma }_{e}+{\gamma }_{f}+\kappa )+\frac{1}{2}\delta (-{\gamma }_{e}+{\gamma }_{f}+\kappa )]}^{2}\}}^{-1}\mathrm{.}$$

**Figure 3:**
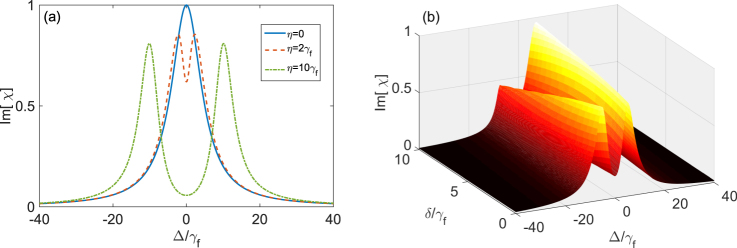
Illustration of the normalized absorption to the probe field in vacuum case with unit *γ*_*f*_. (**a**) shows the imaginary part of the susceptibility *χ* versus the atom-probe detuning Δ when *δ* = 0. The solid, dashed and dash-dotted curves represent different coupling strengths *η* = 0, *η* = 2*γ*_*f*_ and *η* = 10*γ*_*f*_ between the three-level system and the single-mode cavity field. (**b**) shows the imaginary part of the susceptibility *χ* versus the detunings Δ (*δ*) between the probe field (the cavity field) and the three-level system. Here we assume *η* = 5*γ*_*f*_, *γ*_*e*_ = 5*γ*_*f*_ and *κ* = 0.2*γ*_*f*_.

In Fig. 3(a), we show the variation of Im[*χ*] with the detuning Δ when the cavity field resonantly interacts with the three-level system and other parameters are given. The solid, dashed and dash-dotted curves show the absorption spectra for different coupling strengths η. If the three-level system and the cavity field are decoupled (i.e. *η* = 0), as shown in the solid curve in Fig. 3(a), the absorption profile exhibits single absorption peak. The linewidth of the peak is roughly proportional to *γ*_*e*_. Figure 3(a) also shows that the absorption spectra are symmetric when the cavity field resonantly interacts with the three-level system. With the increase of the coupling strength *η*, the absorption profile begins to exhibit a dip. For example, when *η* = 2*γ*_*f*_, there are two peaks and a sharp dip in the center of the absorption spectrum, but the distance between two peaks is smaller than 2*η*. As *η* is further increased to 10*γ*_*f*_, the distance between two peaks becomes into 2*η*.

In Fig. 3(b), Im[*χ*] is further plotted as functions of the detunings Δ and *δ*. We find that the absorption spectrum is asymmetric when the cavity field does not resonantly interact with the three-level system, that is, the asymmetric absorption is due to the detuning between the three-level system and the single-mode cavity field. As shown in Fig. 3(b), the two peaks in the absorption spectrum have the same height when *δ* = 0. With the increase of *δ*, the height of one peak is increased while the other one is decreased, and then presents the asymmetric profile. We note that the two absorption peaks for *δ* < 0 behave oppositely in contrast to those for *δ* < 0. This asymmetric property is very similar to that for a classically driven three-level system^47^ with the ladder-type transition when the driving field does not resonant with the three-level system. By analyzing the imaginary part of the susceptibility *χ*, we find that when *δ* = 0, the expression of Im[*χ*] is an even function of Δ, and has the symmetric resonances. When *δ* ≠ 0, the Δ*δ* term in numerator makes the expression of Im[*χ*] neither an even function nor an odd function of Δ. This results in asymmetric profile.

We mention that the spectrum in^47^ is calculated in the rotating reference frame with the frequency of the control field, the two asymmetric resonances have the same distance relevant to the shifted origin (the origin of the rotating reference frame) when the control field is unresonant with the three-level system, thus two asymmetric resonances have different distances relevant to unshifted origin. This results in an observation that two asymmetric resonances in^47^ are shifted by an unequal amount from the unperturbed resonance at zero probe-detuning. However, in our calculation, we work in the laboratory picture, the two asymmetric resonances have the same distance relevant to the unshifted origin, that is, they have an equal amount frequency shift from the unperturbed resonance at zero probe-detuning.

Below, we will further analyze the reason why there are different distances between two peaks of the absorption spectra in different coupling strengths, as shown in Fig. 3(a). These are related to VIT and vacuum induced ATS.

### Vacuum induced transparency and Autler-Townes splitting

#### Resonance decomposition

In analogy to ATS and EIT for a classically driven three-level system, we further analyze physical mechanism of the dip and two peaks as shown in Fig. 3 when the three-level system is coupled to the single-mode cavity field in vacuum case. Here, we study the condition to discern VIT from vacuum induced ATS. To simplify discussions, we only consider the case that the cavity field resonantly interacts with the three-level system, i.e., *δ* = 0. Following the method as in^8,11^, we first decompose the linear susceptibility *χ* into two resonances. That is, using Eqs (10) and (20), the susceptibility *χ* in vacuum case can be decomposed as

24 $$\chi ={R}_{1}({\rm{\Delta }})+{R}_{2}({\rm{\Delta }})$$

with

$${R}_{1}({\rm{\Delta }})\equiv \frac{\beta }{{{\rm{\Delta }}}_{1}-{{\rm{\Delta }}}_{2}}\frac{{{\rm{\Delta }}}_{1}+i({\gamma }_{f}+\kappa )}{{\rm{\Delta }}-{{\rm{\Delta }}}_{1}},$$

$${R}_{2}({\rm{\Delta }})\equiv \frac{\beta }{{{\rm{\Delta }}}_{1}-{{\rm{\Delta }}}_{2}}\frac{-{{\rm{\Delta }}}_{2}-i({\gamma }_{f}+\kappa )}{{\rm{\Delta }}-{{\rm{\Delta }}}_{2}},$$

and *β* = *μ*_*ge*_/*ε*_0_. Here, we note that the similar decomposition as in Eq. (24) can be obtained for the expression of the susceptibility in^29^ for VIT. That is, the resonance decomposition in Eq. (24) can be applied to analyze the formula in^29^. Hereafter, we call *R*_1_(Δ) and *R*_2_(Δ) as the resonances. The parameters Δ_1_ and Δ_2_ are the complex roots of equation

25 $${{\rm{\Delta }}}^{2}+i({\gamma }_{e}+{\gamma }_{f}+\kappa ){\rm{\Delta }}-C=0,$$

derived from Eq. (20). Here, the parameter *C* is given in Eq. (22) with *δ* = 0. In this case, Δ_1_ and Δ_2_ can be given by

26 $${{\rm{\Delta }}}_{1}=\frac{1}{2}[-i({\gamma }_{e}+{\gamma }_{f}+\kappa )+\sqrt{4{\eta }^{2}-{\eta }_{T}^{2}}],$$

27 $${{\rm{\Delta }}}_{2}=\frac{1}{2}[-i({\gamma }_{e}+{\gamma }_{f}+\kappa )-\sqrt{4{\eta }^{2}-{\eta }_{T}^{2}}],$$

with *η*_*T*_ = |*γ*_*f*_ + *κ* − *γ*_*e*_|. It is clear that both Δ_1_ and Δ_2_ are pure imaginary numbers when $4{\eta }^{2}-{\eta }_{T}^{2} < 0$, but they are complex numbers when $4{\eta }^{2}-{\eta }_{T}^{2} > 0$. Below we further analyze how Δ_1_ and Δ_2_ change with the variations of different parameters.

**Figure 4:**
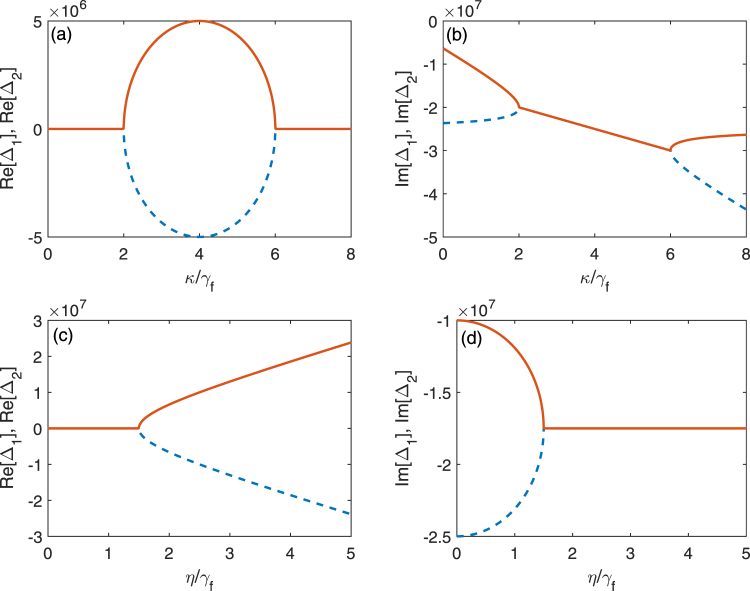
The real and the imaginary parts of Δ_1_ (red solid) and Δ_2_ (blue dashed) in (**a**) and (**b**) are plotted as the function of the cavity decay rate *κ*. Here, we assume *γ*_*e*_ = 5*γ*_*f*_ and *η* = 4*γ*_*f*_. The real and the imaginary parts of Δ_1_ (red solid) and Δ_2_ (blue dashed) in (**c**) and (**d**) are plotted as the function of the coupling strength *η*. Here, we assume *γ*_*e*_ = 5*γ*_*f*_ and *κ* = *γ*_*f*_.

In Fig. 4(a,b), the real and imaginary parts of Δ_1_ and Δ_2_ are plotted as the function of the cavity decay rate *κ* for given parameters, e.g., *η* = 4*γ*_*f*_ and *γ*_*e*_ = 5*γ*_*f*_. From Fig. 4(a,b), we find that $4{\eta }^{2}-{\eta }_{T}^{2}\mathrm{ < 0}$ when *κ* < 2*γ*_*f*_ or *κ* > 6*γ*_*f*_. Both Δ_1_ and Δ_2_ are pure imaginary numbers, and therefore their real parts are zero. However, $4{\eta }^{2}-{\eta }_{T}^{2} > 0$ when the decay rate *κ* of the cavity field is in the range 2*γ*_*f*_ < *κ* < 6*γ*_*f*_, Δ_1_ and Δ_2_ become complex numbers. In this case their real parts have different signs and the same amplitude, but their imaginary parts have the same amplitude.

In Fig. 4(c,d), the real and the imaginary parts of Δ_1_ and Δ_2_ are plotted as the function of the coupling strength *η* between the cavity field and the three-level system for given parameters, e.g., *κ* = *γ*_*f*_ and *γ*_*e*_ = 5*γ*_*f*_. This plot is similar to that in^49^. We find that there are two different parameter regimes: (i) the strong coupling regime when $\eta  > {\eta }_{T}/2$ and (ii) the weak coupling regime when *η* < *η*_*T*_/2. In the weak coupling regime, Δ_1_ and Δ_2_ are pure imaginary numbers with different amplitudes, this means that the resonances *R*_1_(Δ) and *R*_2_(Δ) have the same frequency but different linewidths. In the strong coupling regime, the real parts of Δ_1_ and Δ_2_ have different signs but the same amplitude. This means that the resonances *R*_1_(Δ) and *R*_2_(Δ) have the same linewidth and different frequencies. This property is quite similar to that of PT-symmetric system^31–33^. In the broken PT-symmetric regime, the two coupled modes have different linewidths while the frequencies are degenerate, this corresponds to the weak coupling regime here for *η* < *η*_*T*_/2. In the PT-symmetric regime, the two modes have the same linewidth while the frequencies are different, this corresponds to the strong coupling regime here for *η* > *η*_*T*_/2.

#### Threshold condition for VIT and vacuum induced ATS

To study the condition for realizing VIT and vacuum induced ATS, we should first find the dip in the absorption spectrum. For convenience, we call it as the dip condition. A dip in the absorption spectrum means that the second derivative of Im[*χ*] at Δ = 0 is positive. By solving ${\partial }^{2}{\rm{Im}}\,[\chi ]/\partial {{\rm{\Delta }}}^{2} > 0$, we obtain the dip condition

28 $$\eta  > ({\gamma }_{f}+\kappa )\sqrt{\frac{{\gamma }_{f}+\kappa }{\mathrm{2(}{\gamma }_{f}+\kappa )+{\gamma }_{e}}}.$$

Besides the dip condition, we would like to find a critical value to distinguish VIT and vacuum induced ATS. By applying $4{\eta }^{2}-{\eta }_{T}^{2}=0$, we obtain the threshold

29 $$\eta =\frac{1}{2}{\eta }_{T}\mathrm{.}$$

It is clear that VIT and vacuum induced ATS can be distinguished from this threshold condition. According to^11^, the vacuum induced ATS should occur in the regime $\eta  > {\eta }_{T}\mathrm{/2}$, which is called the strong-coupling regime in analogy to the strong driving regime for the ATS in classically driven three-level system. In this regime, two resonances have different frequencies and the same linewidth as shown in Fig. 4. While VIT should occur in the regime *η* < *η*_*T*_/2, which is called the weak-coupling regime in analogy to the weak driving regime for EIT in classically driven three-level system. In this regime, two resonances have different linewidths and the same frequency as shown in Fig. 4. Therefore, these two coupling regimes can be distinguished by observing the shapes of Im[*R*_1_(Δ)] and Im[*R*_2_(Δ)]. In vacuum induced ATS regime, both Im[*R*_1_(Δ)] and Im[*R*_2_(Δ)] are positive Lorentzian shapes centered at ${\rm{\Delta }}=\sqrt{4{\eta }^{2}-{\eta }_{T}^{2}}$ and ${\rm{\Delta }}=-\sqrt{4{\eta }^{2}-{\eta }_{T}^{2}}$, respectively. While in VIT regime, Im[*R*_1_(Δ)] and Im[*R*_2_(Δ)] have Lorentzian shapes with opposite signs centered at Δ = 0.

Combining the dip condition in Eq. (28) and the threshold condition in Eq. (29), we obtain the condition to realize VIT

30 $$({\gamma }_{f}+\kappa )\sqrt{\frac{{\gamma }_{f}+\kappa }{\mathrm{2(}{\gamma }_{f}+\kappa )+{\gamma }_{e}}} < \eta  < \frac{1}{2}{\eta }_{T},$$

and that to realize vacuum induced ATS

31 $$\eta  > \,{\rm{\max }}\{\frac{1}{2}{\eta }_{T},\,({\gamma }_{f}+\kappa )\sqrt{\frac{{\gamma }_{f}+\kappa }{\mathrm{2(}{\gamma }_{f}+\kappa )+{\gamma }_{e}}}\},$$

for the coupling strength *η*. Note that, if VIT regime exists, the relation $({\gamma }_{f}+\kappa )\sqrt{({\gamma }_{f}+\kappa )/\mathrm{[2(}{\gamma }_{f}+\kappa )+{\gamma }_{e}]} < {\eta }_{T}/2$ should also be satisfied, which leads to

32 $${\gamma }_{e} > \mathrm{2(}{\gamma }_{f}+\kappa \mathrm{).}$$

To simplify the notation, we introduce two parameters *γ*_*R*_ and *η*_*R*_ to interpret the conditions in Eqs (30–32). Here, we define *γ*_*R*_ = *γ*_*e*_/(*γ*_*f*_ + *κ*) and *η*_*R*_ = *η*/(*γ*_*f*_ + *κ*). Using *γ*_*R*_ and *η*_*R*_, Eqs (30–32) can be rewritten as

33 $${\eta }_{d} < {\eta }_{R} < {\eta }_{c},$$

34 $${\gamma }_{R} > \mathrm{2,}$$

for VIT and

35 $${\eta }_{R} > {\eta }_{c},{\gamma }_{R} > \mathrm{2,}$$

36 $${\eta }_{R} > {\eta }_{d},\,{\gamma }_{R} < \mathrm{2,}$$

for vacuum induced ATS with

37 $${\eta }_{d}=\frac{1}{\sqrt{2+{\gamma }_{R}}},$$

38 $${\eta }_{c}=\frac{1}{2}|1-{\gamma }_{R}|\mathrm{.}$$

From above derivations, we conclude that the condition to realize VIT and vacuum induced ATS is similar to that of EIT and ATS, but there are differences. In both VIT and vacuum induced ATS, the dip condition not only depends on the atomic decay rates *γ*_*f*_ and *γ*_*e*_, but also the decay rate *κ* of the cavity, which can be found from Eq. (28). The condition for VIT is ${\gamma }_{e} > \mathrm{2(}{\gamma }_{f}+\kappa )$, however the condition for EIT is ${\gamma }_{e} > 2{\gamma }_{f}$, thus VIT is more difficult to be realized in comparing with EIT.

**Figure 5:**
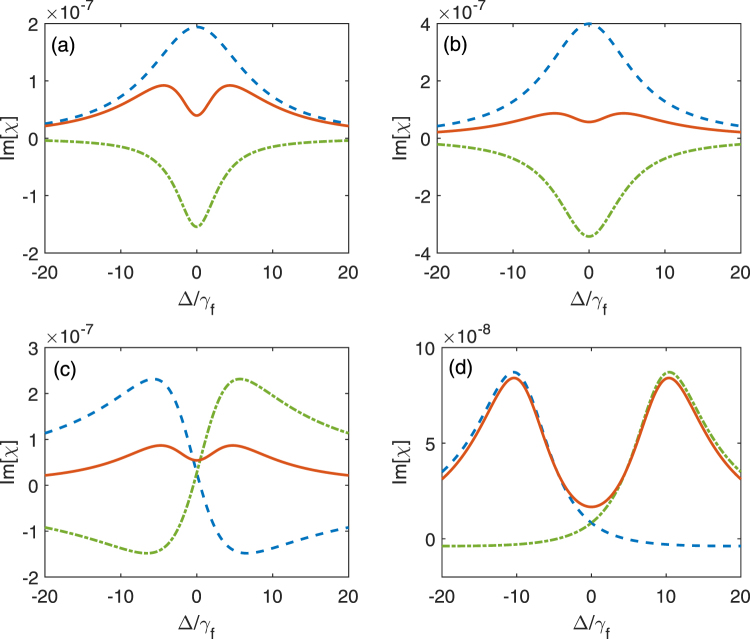
Imaginary parts of the susceptibility Im[*χ*] (red solid curve), the resonances Im[*R*_1_(Δ)] (blue dashed curve) and Im[*R*_2_(Δ)] (green dash-dotted curve) are plotted as the function of the detuning Δ between the three-level system and the probe field for different parameters: (**a**) *γ*_*e*_ = 10*γ*_*f*_, *κ* = 0 and *η* = 3.9*γ*_*f*_; (**b**) *γ*_*e*_ = 10*γ*_*f*_, *κ* = *γ*_*f*_ and *η* = 3.9*γ*_*f*_; (**c**) *γ*_*e*_ = 10*γ*_*f*_, *κ* = *γ*_*f*_ and *η* = 4.1*γ*_*f*_; (**d**) *γ*_*e*_ = 10*γ*_*f*_, *κ* = *γ*_*f*_ and *η* = 10*γ*_*f*_.

In Fig. 5, we show the imaginary part Im[*χ*] of the susceptibility *χ* for absorption spectrum, the resonances Im[*R*_1_(Δ)] and Im[*R*_2_(Δ)] for several sets of possible values of the parameters. Compared to conventional EIT, the cavity decay *κ* here plays a crucial role in realizing VIT. To highlight the effect of *κ*, we assume *γ*_*f*_ is much smaller than *γ*_*e*_ and *η*, this condition may be satisfied in certain physical systems. According to the criteria in Eqs (33–36), we can verify that Fig. 5(a,b) are in VIT regime, while Fig. 5(c,d) are in vacuum induced ATS regime. Comparing Fig. 5(a) (where *κ* = 0) with Fig. 5(b) (where *κ* = *γ*_*f*_), we know that the cavity decay *κ* has a negative effect in VIT as we discussed before. In Fig. 5(b,c), we show the curves of Im[*χ*] for *η* = 3.9*γ*_*f*_ and *η* = 4.1*γ*_*f*_, but other parameters are the same. We can find the transition from VIT in Fig. 5(b) to vacuum induced ATS in Fig. 5(c) with a change of the coupling strength *η*. In Fig. 5(d), the coupling strength is further increased and two Lorentzian peak appear. The curve for Im[*χ*] is completely in vacuum induced ATS regime. We mention that Akaike’s information criterion has been proposed as an objective test to discern the best model for experimentally obtained absorption or transmission spectra^10^ when the data are inconclusive. This criterion can also be applied to analyze the experimental data of VIT and vacuum induced ATS.

We note that our study here is very different from those in^23,24^, where they studied EIT and ATS using three energy levels chosen from many energy levels of a two-level system dressed by a cavity field. In their studies, both the classical control and probe fields are applied to the selected three-level system. The role of the cavity field in^23,24^ is to assist the two-level system realizing a three-level system. These systems in^23,24^ are still used to study a conventional EIT and ATS. However, we here study a three-level system, in which two upper energy levels are coupled to a quantized single-mode cavity field, one and only one classical probe field is applied to the system. The quantized cavity field acts as a control field. Thus we study quantized-field-induced quantum interference and frequency shift, we here call them as VIT and vacuum induced ATS. Therefore, the cavity field in our study here and in^23,24^ has very different purpose.

### Photon resolved Autler-Townes splitting

In this section, we further study the absorption when the quantized control field contains the finite number of photons at the steady state. This situation is different from the classical control field with very large number of photons for EIT and ATS, also different from the quantized control field without photon for VIT and vacuum induced ATS. The finite photon number of quantized control field might be realized by incoherent pumping or coherent pumping to the quantized control field.

### Incoherent pumping

We assume that the incoherent pumping is realized by taking the environmental temperature *T* into account. In this case, the master equation in Eq. (12) is further modified to

39 $$\begin{array}{rcl}\dot{\rho } & = & \frac{1}{i\hslash }[H,\rho ]+({n}_{th}+\mathrm{1)}\kappa \mathrm{(2}a\rho {a}^{\dagger }-{a}^{\dagger }a\rho -\rho {a}^{\dagger }a)\\  &  & \,+{n}_{th}\kappa \mathrm{(2}{a}^{\dagger }\rho a-a{a}^{\dagger }\rho -\rho a{a}^{\dagger })+\sum _{i,j=e,f,g}^{i\geqslant j}\frac{{\gamma }_{ij}}{2}\mathrm{[2}{\sigma }_{ji}\rho {\sigma }_{ij}-{\sigma }_{ii}\rho -\rho {\sigma }_{ii}]\end{array}$$

with the thermal photon ${n}_{th}=1/({e}^{\hslash {\omega }_{c}/{k}_{b}T}-\mathrm{1)}$ of the quantized control field. Here, for simplicity and without loss of generality, we have neglected effect of the temperature on the three-level system.

**Figure 6:**
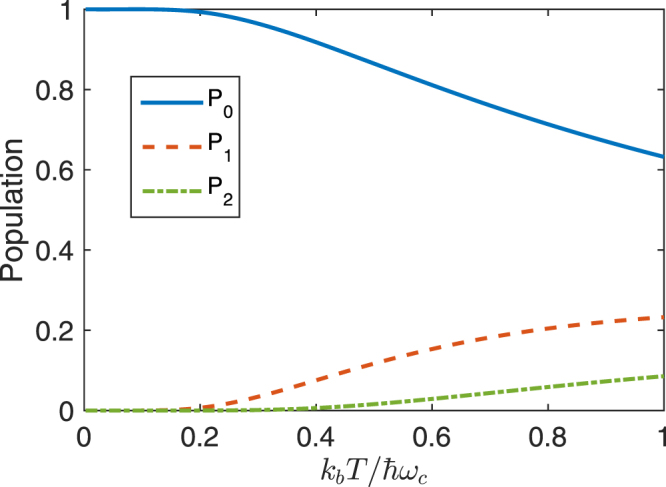
The populations in the states |0, *g*〉, |1, *g*〉 and |2, *g*〉 as a function of ${k}_{b}T/\hslash {\omega }_{c}$ when the system reaches the steady state. Here, we assume that the three-level system resonantly interacts with the quantized control field, i.e., *δ* = 0. *P*_0_ (blue solid curve), *P*_1_ (red dashed curve) and *P*_2_ (green dash-dotted curve) represent the population in |0, *g*〉, |1, *g*〉 and |2, *g*〉, respectively. Here we assume *γ*_*e*_ = 5*γ*_*f*_, *κ* = 0.2*γ*_*f*_ and *η* = 2*γ*_*f*_, that is, we use *γ*_*f*_ as units in our numerical calculations.

At the finite temperature, *n*_*th*_ ≠ 0, thus not all population remains in the ground state |0,*g*〉 when the system reaches steady state, other states, e.g., the states |1, *g*〉, |2, *g*〉, $\cdots$, |*n*, *g*〉, may also be occupied. That is, all possible states of the system might be involved in zero-order solutions of all matrix elements of the density matrix. Therefore, it is very difficult to obtain analytical solutions of *χ* at the finite temperature. From Eq. (10), we know that the susceptibility *χ* is only related to states |*n*, *g*〉 and |*n*, *e*〉. To observe how the thermal photon affects the population in different states, we numerically^50^ solve Eq. (39) by truncating photon number to 60. In Fig. 6, we show how the populations in, e.g., the states |0, *g*〉, |1, *g*〉 and |2, *g*〉, vary with the environmental temperature for *ε* = 0 when the system reaches the steady state under the resonant interaction between the three-level system and the quantized control field. As shown in Fig. 6, when the environmental temperature is low, almost all population is in the ground |0, *g*〉, the population in other states is negligibly small. However, with the increase of the temperature, the population in the state |0, *g*〉 is decreased, the populations in other states, e.g., |1, *g*〉 and |2, *g*〉, are increased.

**Figure 7:**
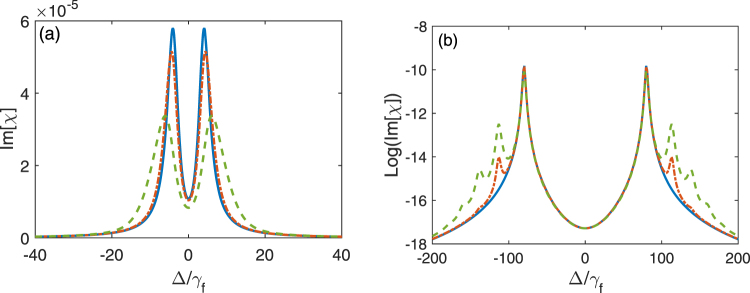
The imaginary part Im[*χ*] of the susceptibility *χ* is plotted as the function of the detuning Δ between the three-level system and the probe field when the three-level system resonantly interacts with the quantized control field. (**a**) shows the absorption spectra in the weak coupling regime, e.g., *η* = 4*γ*_*f*_. (**b**) shows the absorption spectra in the strong coupling regime, e.g., *η* = 80*γ*_*f*_. In each figure, we take three different temperatures: ${k}_{b}T/\hslash {\omega }_{c}=0$ (blue solid curve), ${k}_{b}T/\hslash {\omega }_{c}=0.25$ (red dash-dotted curve), ${k}_{b}T/\hslash {\omega }_{c}=0.5$ (green dashed curve). Here, we assume *γ*_*e*_ = 5*γ*_*f*_, *κ* = *γ*_*f*_.

Using master equation in Eq. (39), Im[*χ*] is plotted as a function of the detuning Δ in Fig. 7 under the resonant interaction (i.e., *δ* = 0) between the three-level system and the quantized control field. Figure 7 shows Im[*χ*] versus Δ when the three-level system and the quantized field are either in weak or strong coupling regime for VIT or vacuum induced ATS with the rescaled environmental temperature ${k}_{b}T/\hslash {\omega }_{c}=\mathrm{0,0.25}$ and 0.5, respectively. Here, we take a high temperature to observe the effect of the thermal photons on the spectrum. At the zero temperature, the quantized control field is in vacuum when the whole system reaches steady state, thus Im[*χ*] is only proportional to 〈*e*, 0|*ρ*|0, *g*〉. Under certain condition, two peaks can appear in the absorption spectra as shown in Figs 3 and 5. At the finite temperature, there are populations in the states |*n*, *g*〉 with *n* > 0. Thus, Im[*χ*] should include not only 〈*e*, 0|*ρ*|0, *g*〉 for vacuum but also 〈*e*, *n*|*ρ*|*n*, *g*〉 with *n* > 0 for the finite photon number. In the weak coupling regime for VIT, the distances between any two peaks of different photon number in the same side of the spectrum are smaller than the linewidth of each peak, thus we cannot observe photon number resolved peaks. However, in the strong coupling regime for vacuum induced ATS, the distances between any two peaks of different photon numbers in the same side of the spectrum can be larger than the linewidth of each peak, thus photon resolved peaks can be observed. For example, two peaks approximately locate at −*η* and *η* in the spectrum for vacuum case corresponding to 〈*e*, 0|*ρ*|0, *g*〉, but two peaks locate at $-\eta \sqrt{2}$ and $\eta \sqrt{2}$ in the spectrum for single-photon case corresponding to 〈*e*, 1|*ρ*|1, *g*〉. When ($(\sqrt{2}-1)\eta  > {\gamma }_{e}$, single-photon peak and vacuum peak can be resolved.

Figure 7(a) shows Im[*χ*] versus Δ in the weak coupling regime for VIT. When the temperature of the system is not high, Im[*χ*] for the finite photon number at finite temperature almost overlaps with that for vacuum at zero temperature, but the heights of two peaks in absorption spectrum are slightly reduced due to the effect of the temperature. This is because only single occupation plays a role, other occupations are negligibly small. When the temperature is further increased, as shown in Fig. 6, the more states |*n*, *g*〉 (*n* > 0) for higher energy levels are involved. Although Im[*χ*] is proportional to the summation of all possible matrix elements 〈*e*, *n*|*ρ*|*n*, *g*〉 as shown in Eq. (17), the photon-number dependent peaks in absorption spectrum are not resolved in the weak coupling regime. As a result, (i) the linewidths of two peaks are broadened and the heights of two peaks are suppressed; (ii) the position of each peak is shifted comparing with that at zero temperature, because the peak of each component corresponding to 〈*e*, *n*|*ρ*|*n*, *g*〉 in Im[*χ*] is slightly different, the peak position of the summation for all these peaks is different from anyone of these peaks. Such photon number dependent EIT might be used to realize photon number dependent group delay for the probe field. However, in the strong coupling case, as shown in Fig. 7(b), when the temperature of the system is zero, Im[*χ*] exhibits two peaks, we call them as vacuum induced ATS. When the temperature of the system is further increased, Im[*χ*] exhibits even number of peaks, we call them as photon number resolved Autler-Townes spectrum. But if the temperature is further increased, the linewidth of each peak also becomes larger, then many peaks for photon number resolved spectrum will gradually become into two peaks.

### Coherent pumping

We now consider another case that the quantized control field is pumped by a weak coherent field. In this case, the Hamiltonian in Eq. (1) is modified to

40 $${H}_{{\rm{Coh}}}=\hslash {\omega }_{c}{a}^{\dagger }a+\hslash {\omega }_{e}|e\rangle \langle e|+\hslash {\omega }_{f}|f\rangle \langle f|+\hslash \eta (|e\rangle \langle f|a+{\rm{h}}{\rm{.c}}{\rm{.}})+\hslash \varepsilon (|e\rangle \langle g|{e}^{-i{\omega }_{p}t}+{\rm{h}}{\rm{.c}}\mathrm{.)}+\hslash {\rm{\Omega }}({a}^{\dagger }{e}^{-i{\omega }_{d}t}+{\rm{h}}{\rm{.c}}{\rm{.}}),$$

where *ω*_*d*_ is the frequency of the pumping field and Ω is the coupling strength between the pumping field and quantized control field. Similar to the effect of thermal photons, a coherent pumping field can also modify the occupations of photons in different states when the whole system reaches the steady state. This will also result in photon number dependent absorption to the probe field. To solely consider the pumping effect and without loss of generality, we assume that the whole system is at zero temperature. In this case, replacing *H* in Eq. (12) by *H*_Coh_ in Eq. (40), we can numerically study the absorption spectrum by solving the master equation. In our simulation, the photon number is truncated to 60.

**Figure 8:**
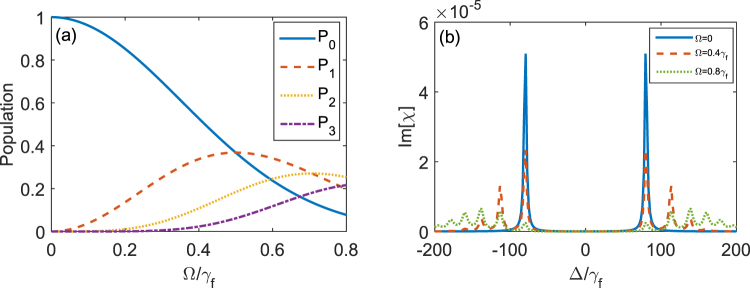
(**a**) The populations in the states |0, *g*〉, |1, *g*〉, |2,*g*〉 and |3, *g*〉 as a function of the driving strength Ω when the system reaches the steady state. Here, we assume that the three-level system resonantly interacts with the quantized control field, i.e., *δ* = 0. *P*_0_ (blue solid curve), *P*_1_ (red dashed curve), *P*_2_ (yellow dotted curve), *P*_3_ (purple dash-dotted curve) represent the populations in |0, *g*〉, |1, *g*〉, |2, *g*〉 and |3, *g*〉, respectively. (**b**) The imaginary part Im[*χ*] of the susceptibility *χ* versus the detuning Δ. Here we assume *γ*_*e*_ = 5*γ*_*f*_, *κ* = *γ*_*f*_ and *η* = 80*γ*_*f*_, that is, we use *γ*_*f*_ as units in our numerical calculations.

As shown in Fig. 8(a), all the population remains in the ground |0, *g*〉 when the pumping field is not applied, i.e., Ω = 0. With the increase of the strength Ω of the pumping field, the population occupation in the state |0, *g*〉 is decreased while the populations in the states |1, *g*〉, |2, *g*〉 and |3, *g*〉 are increased. In Fig. 8(b), we show the imaginary part Im[*χ*] of the susceptibility *χ* versus the detuning Δ when the three-level system and the quantized control field is in the strong coupling regime for vacuumed induced ATS. When Ω = 0, as shown in the blue solid curve in Fig. 8(b), the absorption spectrum corresponds to vacuum induced ATS. When Ω = 0.4*γ*_*f*_, as shown in the red dashed curve in Fig. 8b, the peaks located at Δ = ±*η*, $\pm \sqrt{2}\eta$ and $\pm \sqrt{3}\eta$ emerge. The heights of these peaks correspond to the populations in the states |0, *g*〉, |1, *g*〉, |2, *g*〉 and |3, *g*〉 in Fig. 8(a) at Ω = 0.4*γ*_*f*_. When Ω = 0.8*γ*_*f*_, the population in the state |0, *g*〉 is smaller than that in each of the states |1, *g*〉, |2, *g*〉 and |3, *g*〉, and the corresponding peaks located at Δ = ±*η* are also lower than others.

We find that the absorption spectrum of the probe field in coherent pumping is very similar to that of the incoherent pumping when the coupling strength between the three-level system and the quantized control field is in the parameter regime of VIT. That is, there might be two peaks and one dip in the absorption spectrum. With the increase of the strength of the pumping field, the heights of two peaks are reduced and the linewidths of two peaks are broadened. When the strength of the pumping field is further increased, the quantized control field approaches to the classical one, then the spectrum approaches to that of EIT. There is a difference for the absorption spectra between the coherent pumping and the incoherent pumping. When the quantized control field is coherently pumped, the steady-state population in the state $|n,\,g\rangle$ (*n* ≠ 0) can exceed that in the state $|0,\,g\rangle$ when the strength Ω of the pumping field is large enough. If the coupling strength between the three-level system and the quantized control field is in the parameter regime of vacuum induced ATS, as shown in Fig. 8(b) for the strong pumping case (the green dotted curve), then we have photon number resolved spectrum. The peaks located in Δ = ±*η* correspond to the transition from $|0,\,g\rangle$ to $|0,\,e\rangle$. The peaks located in ${\rm{\Delta }}=\pm \sqrt{n}\eta$ correspond to the transition from $|n,g\rangle$ to $|n,e\rangle$ (*n* ≠ 0). From Fig. 8(a), we can find that the steady-state population in the state $|0,\,g\rangle$ is smaller than that in the state $|n,g\rangle$ (*n* ≠ 0) when Ω = 0.8*γ*_*f*_. This makes that the heights of peaks located in Δ = ±*η* are lower than those of peaks located in ${\rm{\Delta }}=\pm \sqrt{n}\eta$. However, when the quantized control field is incoherently pumped, the steady-state population in the state $|0,\,g\rangle$ is always larger than those in other states $|n,g\rangle$ (*n* ≠ 0). Such difference between the coherent and incoherent pumping is due to the statistical distributions for coherent and thermal states^59^.

## Discussions on possible applications to superconducting quantum circuits

Our study here can in principle be applied to any Λ-type three-level system, coupled to a quantized single-mode cavity field and detected by a weak probe field, as schematically shown in Fig. 1. For example, a three-level atomic system, coupled to a quantized field^29,51^, is good for demonstrating VIT because of smaller decay rate of the first excited state and weaker coupling strength between the three-level system and quantized control field, but atomic systems might not be easy to demonstrate vacuum induced ATS because the coupling strength between the three-level system and the quantized control field is not very strong.

Let us now explore another possibility to demonstrate these phenomena using superconducting circuit QED system^34^, which is extensively studied for quantum information processing and quantum optics on superconducting chip^18–20^. For concreteness of discussions, we assume that the three-level system with Λ-type transitions is constructed by a superconducting flux qubit circuit^52–54^ when the magnetic flux bias deviates from the optimal point. Recently, such a three-level flux qubit circuit is used to demonstrate the correlated microwave lasing^55^ by coupling it to two modes of a coplanar waveguide resonator. Using experimentally accessible parameters in^55^, e.g., decay rates *γ*_*e*_ = 2*π* × 7.5 MHz and *γ*_*f*_ = 2*π* × 3.25 MHz of three-level system, and decay rate *κ* = 2*π* × 0.63 MHz for one of modes, and coupling strength *η* = 2*π* × 36 MHz between this mode and three-level system, we can obtain *γ*_*R*_ = 1.93, *η*_*R*_ = 9.28, *η*_*d*_ = 0.50 and *η*_*c*_ = 0.47. Evaluating the parameters *γ*_*R*_, *η*_*R*_, *η*_*d*_ and *η*_*c*_ with the conditions in Eqs (33–36), we find that the vacuum induced ATS can be demonstrated using this set of experimental parameters. Moreover, it is possible to demonstrate photon number resolved ATS in this system. We also find that the value of *γ*_*R*_, with parameters in^55^, is very close to 2. Thus, if the experimental parameters can be further optimized so that *γ*_*R*_ > 2, and also a proper coupling strength *η* can be chosen, then VIT can be realized in such superconducting three-level system coupled to a single mode microwave field. We note that the vacuum induced Autler-Townes doublet^56^ has been experimentally realized in the circuit QED system recently.

Although we only discuss possible realization in a three-level superconducting flux qubit circuit which is coupled to a microwave cavity field, the study here can also be applied to phase^37,38,57^ and other superconducting quantum circuits, which possess Λ-type transitions. We mention that the inversion symmetry of the potential energy for superconducting flux^52–54^, transmon^58,59^ and Xmon^60^ qubit circuits is well defined, thus the transition from the ground state to the second excited state is forbidden at the optimal point. They have ladder-type transitions and no Λ-type transitions at the optimal point. How VIT and vacuum induced ATS occur in such a ladder-type three-level system is still under study.

## Conclusions

In conclusion, we have studied the absorption spectrum of a probe field by a Λ-type three-level system, in which two upper energy levels are coupled to a quantized single-mode control field. If the quantized control field is replaced by a classical control field, then the system is usually studied for EIT and ATS. We find that there are similarities and differences in the absorption spectra for the classical and quantized control field. (1) If the quantized control field is in vacuum, then the vacuum induced absorption spectrum is very similar to EIT or ATS spectrum. That is, there is a transparency windows formed by two peaks and a dip in the absorption spectrum. In the parameter regime of the weak coupling between the quantized control field and three-level system, VIT might occur. Similar to EIT, the distance between two peaks in the absorption spectrum for VIT is smaller than two times of the coupling strength. Moreover, we find that VIT is more difficult to be realized than EIT when the cavity leakage is included. That is, the cavity decay plays a negative role in the realization of VIT. In the strong coupling regime, vacuum induced ATS occurs. Similar to ATS, the distance between two peaks in the absorption spectrum for vacuum induced ATS is two times of the coupling strength. (2) If the quantized control field contains finite number of photons, then in the weak coupling regime, absorption spectrum is also similar to that of EIT. Only difference is that the heights of two peaks will be suppressed and the linewidths of two peaks are broadened with the increase of the photon number. When photon number is further increased, the quantized control field approaches classical one, then the spectrum approaches to that of EIT. In particular, in the strong coupling regime, we find the so-called photon number resolved ATS, which is very different from ATS. There are even number of peaks in the absorption spectrum. We mention that there were experiments on photon number resolved spectrum^61,62^ when a superconducting qubit (a two-level system) is strongly coupled to a quantized field. There, two-level system is dispersively coupled to the quantized field, the photon number is observed by virtue of the ac Stark shifted qubit frequency^61,62^. Here, a three-level system is resonantly coupled to the quantized field, the photon number is observed by virtue of the resonant absorption spectrum.

Comparing with studies for VIT^28,29^, we here give the threshold condition to discern VIT from vacuum induced ATS in such a system. The realization of VIT requires that the coupling strength between the three-level system and the quantized control field is smaller than a critical value, which depends on the damping rates of the three-level system and the quantized control field. However, the realization of the vacuum induced ATS requires that the coupling strength between the three-level system and the quantized control field is larger than the critical value. We also show that the parameter changing from VIT to vacuum induced ATS is very similar to that from broken *PT* symmetry to *PT* symmetry. Furthermore, we studied the photon number dependent spectrum, in particular, we show a photon number resolved ATS in the parameter regime of the strong coupling between the quantized control field and three-level system.

We also explore possible experiments using natural atomic systems or superconducting quantum circuits. We find that three-level natural atomic systems might be a good candidate to demonstrate VIT because of smaller decay rate of the first excited state and weaker coupling strength between three-level system and quantized control field, but atomic systems might not be easy to demonstrate vacuum induced ATS because the coupling strength between the three-level system and the quantized control field is not very strong. However, vacuum induced ATS is easy to be demonstrated in the superconducting quantum circuits because the coupling strength between three-level superconducting qubit circuit and the quantized control field can be very strong, but VIT may not be easy to be demonstrated. Thus, to show VIT in superconducting quantum system, the decay rates of two excited states of three-level system should be further optimized.

In summary, we study the quantized field controlled absorption spectrum in a three-level system. In particular, we give a threshold to discern VIT from vacuum induced ATS. We also find photon number resolved ATS, which is very different from ATS and vacuum induced ATS. Our finding has potential applications. For example, using the photon number resolved ATS, the photon statistics inside the cavity can be distinguished even the cavity field resonantly interacts with the three-level system. In^61^, the photon statistics is distinguished by dispersive interaction between two-level system and cavity field. However, VIT with different photon numbers inside the cavity can result in different group velocity delay, which might be used for photon number filter^30^. We hope that our study can motivate more experiments to realize photon control for weak probe field at single-atom and single-photon level.
