# Publisher Correction: Vacuum induced transparency and photon number resolved Autler-Townes splitting in a three-level system

**DOI:** 10.1038/s41598-018-31111-3

**Published:** 2018-08-22

**Authors:** Jiang-hao Ding, Sai-nan Huai, Hou Ian, Yu-xi Liu

**Affiliations:** 1 Institute of Microelectronics, Tsinghua University, Beijing, 100084 China; 2 Institute of Applied Physics and Materials Engineering, University of Macau, 999078 Macau, China; 3 Tsinghua National Laboratory for Information Science and Technology (TNList), Beijing, 100084 China

Correction to: *Scientific Reports* 10.1038/s41598-018-22666-2, published online 14 March 2018

In Figure 1, the schematic diagram is incorrect. The correct Figure 1 appears below as Figure 1.

**Figure 1:**
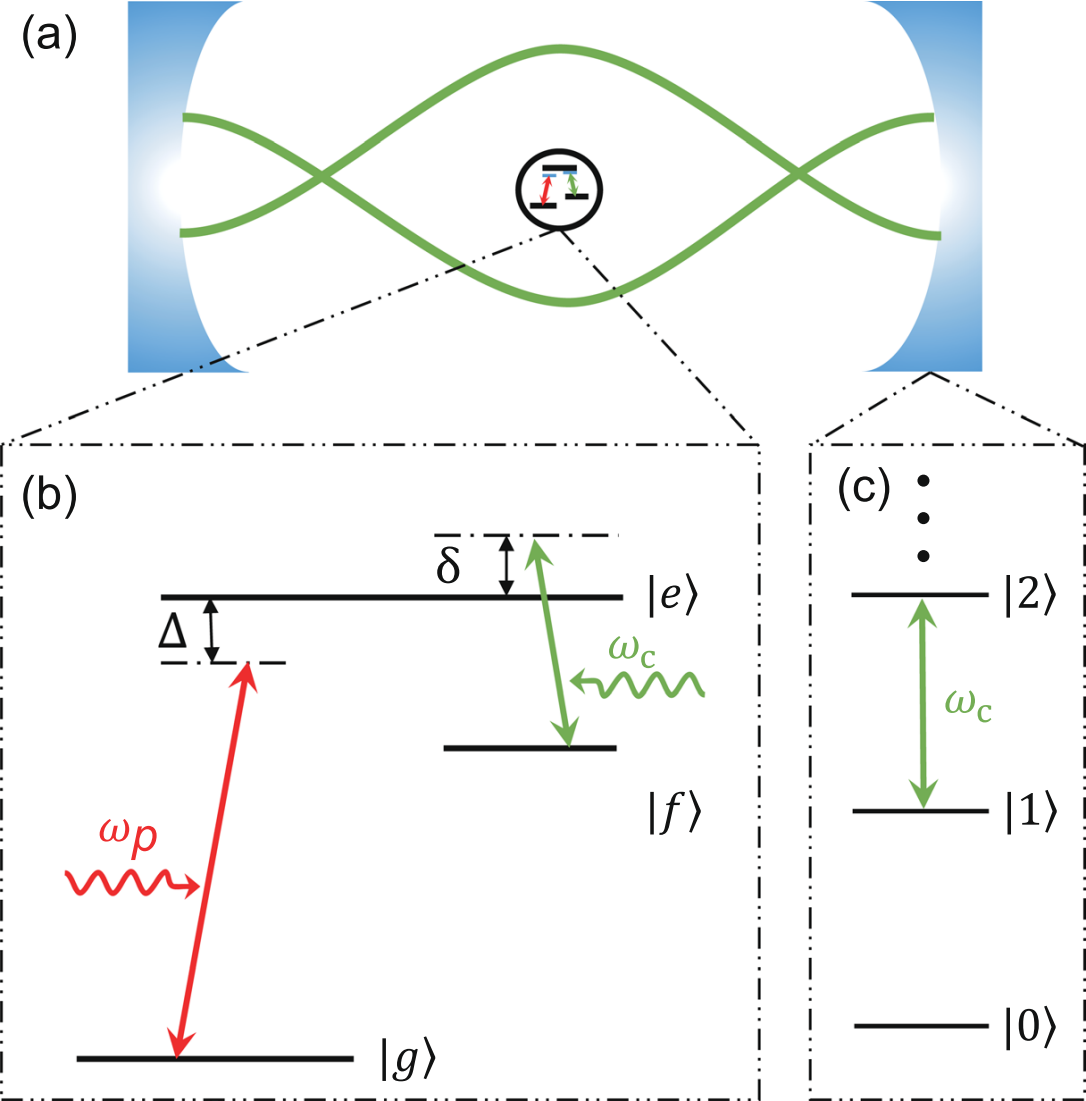
(**a**) A schematic diagram for a three-level system with Λ-type transitions inside the cavity. Here two green curves schematically represent the cavity field. (**b**) The schematic diagram for three-level system coupled to a single-mode cavity field and a classical probe field. The cavity field induces the transition between the energy levels |*e*〉 and |*f*〉, however the probe field induces the transition between the energy levels |*e*〉 and |*g*〉. Here, Δ = *ω*_*e*_ − *ω*_*p*_ is the detuning between the frequency ωp of the probe field and the transition frequency *ω*_*e*_ of the three-level system, *δ* = *ω*_*c*_ − (*ω*_*e*_ − *ω*_*f*_) denotes the detuning between the frequency ωc of the cavity field and the transition frequency *ω*_*e*_ − *ω*_*f*_ of the three-level system. (**c**) A schematic diagram for the energy levels of the cavity field with the equal energy levels spacing *ω*_*c*_.

